# Ode to Epileptologists!

**Published:** 2011

**Authors:** Ajith Cherian

**Affiliations:** Sree Chitra Tirunal Institute for Medical Sciences & Technology (SCTIMST), Medical College P.O, Trivandrum - 695 011, Kerala, India. E-mail: drajithcherian@yahoo.com

Confined to an 8 × 8 room
Perceived by technology 24 × 7
I lay there waiting doomed like
The condemned prisoner for hangman’s noose.
Glee they take when my body shakes
Their eyes light up when my torso jerks
I bare it all; nothing to hide
No place for my self-esteem to hide.
Forced I am to void my bowels
Where I sleep and eat and rest
Prison inmates are better off
At least they see sky through bars.
A buzzer I have for all auras
Press and they come to harass me
Questions from all sides CBI like
Poor me! left verbally challenged.
Night I try to get some sleep
Cruelly woken up by a tonic cry
One next room is shaking all over
All I know next is mine.
Light they throw upon my face
It nearly blinds me, I screw my eyes
They continue with some abstract art
Next I pant; dogs put to shame.
After days of ordeal I’m informed
Cryptogenic-refractory-uncertain localization
Greek and Latin its all to me
I’m glad it’s over, I’m free.

